# Regulatory Mechanisms of Transcription Factors in Plant Morphology and Function 2.0

**DOI:** 10.3390/ijms25042010

**Published:** 2024-02-07

**Authors:** Tomotsugu Koyama

**Affiliations:** Bioorganic Research Institute, Suntory Foundation for Life Sciences, Seikacho, Kyoto 619-0284, Japan; koyama@sunbor.or.jp

In plants, gene regulation underlies organ development and responses to environmental changes. Transcription factors (TFs) are the key proteins that bind DNA to target genes and regulate transcription. TFs are classified on the basis of the sequence similarity of their DNA-binding domains, which are evolutionarily conserved in plants [1]. Recently developed genome sequencing technologies have identified several genes that encode such conserved TFs in various plant species. The identification of TF genes in certain plants of interest enables us to understand the regulation of genes that are vital for their development and responses to environmental changes. In this vein, a striking feature of this Special Issue is its focus on a broad range of TFs from Arabidopsis, crops, and wild plant species.

This Special Issue includes functional analyses of the TFs involved in developmental plasticity. The plasticity of organ development is an intriguing aspect of developmental mechanisms. Callus formation in developing organs is associated with dynamic changes in gene expression in response to growth regulators and external stimuli. TFs control the initiation and suppression of callus formation [2]. Plasticity has also been observed during leaf development and senescence. Leaf development underlies important metabolic processes, such as photosynthesis, whereas leaf senescence is associated with cell death and the relocation of nutrients inside a plant [3]. Precocious senescence is harmful to plants; however, accelerated senescence may facilitate the survival of the next generation of plants under stress. Thus, senescence requires a tightly regulated gene network, and TFs play an important role in the regulation of leaf senescence onset. 

This Special Issue also comprises the identification and molecular analyses of TFs responsible for biotic and abiotic stress. Plants respond to continuous changes in environmental conditions, such as dehydration, salinity, and heat. TFs play a central role in the trade-off between the induction of defense genes and repression of growth genes [4]. The integration of stress responses and plant hormone signaling pathways makes it crucial to comprehend how TFs interact with these pathways in a variety of crops and wild plants. A previous report suggested the possibility of a TF gene conferring tolerance to stress conditions in transgenic plants, such as dehydration [5]. Thus, the identification and molecular analysis of TFs in certain plants are important for the future improvement of plant stress tolerance. 

The intriguing research and review papers featured in this Special Issue discuss the recent progress in uncovering the roles of TFs in many aspects of plant development and function. Functional analysis of these TFs is crucial for comprehending their mechanistic action because advances in sequencing technology have made it easier to identify TF genes in a variety of plants. Significant advances have been made in this research area; however, key scientific questions remain to be answered. For example, it is not fully understood how TFs recruit transcriptional machinery into the DNA of target genes or how TFs find the precise DNA of the target genes in the genome [6]. As such, future studies will provide a full perspective of the TFs that regulate gene expression for plant morphogenesis and function.
